# Non-Invasive Monitoring of Temporal and Spatial Blood Flow during Bone Graft Healing Using Diffuse Correlation Spectroscopy

**DOI:** 10.1371/journal.pone.0143891

**Published:** 2015-12-01

**Authors:** Songfeng Han, Michael D. Hoffman, Ashley R. Proctor, Joseph B. Vella, Emmanuel A. Mannoh, Nathaniel E. Barber, Hyun Jin Kim, Ki Won Jung, Danielle S. W. Benoit, Regine Choe

**Affiliations:** 1 Institute of Optics, University of Rochester, Rochester, New York, United States of America; 2 Department of Biomedical Engineering, University of Rochester, Rochester, New York, United States of America; 3 Department of Orthopaedics and Center for Musculoskeletal Research, University of Rochester Medical Center, Rochester, New York, United States of America; 4 Department of Otolaryngology-Head and Neck Surgery, University of Rochester Medical Center, Rochester, New York, United States of America; 5 Department of Chemical Engineering, University of Rochester, Rochester, New York, United States of America; 6 Department of Biomedical Genetics, University of Rochester Medical Center, Rochester, New York, United States of America; 7 Department of Electrical and Computer Engineering, University of Rochester, New York, United States of America; Medical University of South Carolina, UNITED STATES

## Abstract

Vascular infiltration and associated alterations in microvascular blood flow are critical for complete bone graft healing. Therefore, real-time, longitudinal measurement of blood flow has the potential to successfully predict graft healing outcomes. Herein, we non-invasively measure longitudinal blood flow changes in bone autografts and allografts using diffuse correlation spectroscopy in a murine femoral segmental defect model. Blood flow was measured at several positions proximal and distal to the graft site before implantation and every week post-implantation for a total of 9 weeks (autograft n = 7 and allograft n = 10). Measurements of the ipsilateral leg with the graft were compared with those of the intact contralateral control leg. Both autografts and allografts exhibited an initial increase in blood flow followed by a gradual return to baseline levels. Blood flow elevation lasted up to 2 weeks in autografts, but this duration varied from 2 to 6 weeks in allografts depending on the spatial location of the measurement. Intact contralateral control leg blood flow remained at baseline levels throughout the 9 weeks in the autograft group; however, in the allograft group, blood flow followed a similar trend to the graft leg. Blood flow difference between the graft and contralateral legs (Δ*rBF*), a parameter defined to estimate graft-specific changes, was elevated at 1–2 weeks for the autograft group, and at 2–4 weeks for the allograft group at the proximal and the central locations. However, distal to the graft, the allograft group exhibited significantly greater Δ*rBF* than the autograft group at 3 weeks post-surgery (*p* < 0.05). These spatial and temporal differences in blood flow supports established trends of delayed healing in allografts versus autografts.

## Introduction

Every year, more than 500,000 U.S. and 2.2 million global orthopedic procedures are carried out to treat bone defects [1, 2]. Among these procedures, autograft transplantation, which requires endogenous bone and soft tissue transplantation from a non-load bearing region of the skeleton, often achieves complete healing [1, 2]. However, autograft transplantation for critical-sized bone defects is limited by tissue availability and associated donor site morbidity. Therefore, allograft transplantation, using processed cadaveric bone to replace the bone defect, is the “gold-standard” treatment for critical-sized bone defects [1, 3, 4]. To reduce immunogenicity, allografts are completely devitalized; therefore, the periosteum is lost. The periosteum is a thin tissue layer covering the outer bone surface that contains osteoblasts, osteogenic precursor cells, and periosteal stem cells [5]. Studies have shown that the periosteum is crucial in vascularization, bone callus formation, and subsequent healing and remodeling of autografts in critical-sized defect repair [6]. As a result, allografts exhibit poor host osteointegration with a 60%, 10-year post-implantation failure rate [7–9].

Various tissue engineering approaches to improve allograft integration and healing have been developed by exploiting the delivery of periosteum mimetic cells and/or growth factors to the graft site [10–13]. The effectiveness of these new approaches is commonly assessed using an animal model (e.g., murine segmental femoral defect model) by quantifying vascularization and mechanical strength after graft implantation [4, 11–14]. Vascularization is recognized as a critical and tightly regulated process in bone healing, as blood vessels deliver oxygen, nutrients, growth factors, and circulating cells to the graft site [15]. For autografts, it is well established that the progression of healing and remodeling starts with vascular infiltration, followed by osteoclast migration and activation, then osteoblast progenitor cell infiltration [16]. Using lead-chromate vascular perfusion techniques and subsequent micro-computer tomography (microCT) reconstruction [14, 17], host vascular infiltration of autografts was shown to be significantly greater than that of allografts even after 16 weeks post graft surgery [3, 13, 14]. However, this technique cannot provide longitudinal vascular volumes of the same animal due to the requirement of animal sacrifice. Furthermore, overall blood supply to grafts may depend not only on intravascular volume, but also blood flow rate which can change rapidly, especially in existing vasculature of native bones and tissues surrounding grafts, due to inflammation and metabolic demands in the early phase of healing [15]. Non-invasive characterization of longitudinal blood flow changes in different grafts has the potential to not only advance our understanding of graft healing, but to establish standards to compare new tissue-engineered approaches.

Blood flow measurements in bone are often confounded by high bone density, as well as the heterogeneity of the intraosseous blood supply [18]. As a result, few methods are available to measure blood flow in bone, such as radioactive or fluorescent microsphere techniques [19, 20], magnetic resonance imaging (MRI) and positron emission tomography (PET) [18], and laser Doppler flowmetry [21, 22]. However, the application of these techniques for longitudinal studies of blood flow during bone healing has proven difficult due to technical limitations or the cost associated with these methods [18, 23]. Although blood flow at multiple time points can be followed by injection of different isotopes, fluorescent dyes, or fluorescent microspheres, these methods require animal sacrifice and are experimentally difficult [15]. MRI and PET require injection of contrast agents or radiotracers, and may be expensive for longitudinal measurements. Limited penetration depth of laser Doppler technique requires surgical exposure of the bone [21] and, therefore alters the graft site surgically.

Diffuse correlation spectroscopy (DCS) is a non-invasive technique that quantifies microvascular blood flow in deep tissue [24]. It is suitable for longitudinal monitoring studies since no ionizing radiation or contrast agents are used, and the measurement is inexpensive to implement. DCS has been applied to different tissues such as tumors, brain, and skeletal muscle both in animals and humans *in vivo* [24]. Furthermore, it has been validated with other methods for measuring blood flow, such as Doppler ultrasound [25–29], laser Doppler flowmetry [30, 31], arterial-spin labeled MRI [32–35], xenon CT [36], and fluorescent microsphere measurements [37].

In this contribution, we present longitudinal blood flow measurements during the first 9 weeks of graft healing in a murine segmental femoral graft model using diffuse correlation spectroscopy. Our results show that autograft and allograft groups exhibit different spatial and temporal patterns in blood flow during graft healing. To our knowledge, this study is the first comparison of weekly longitudinal blood flow patterns in autografts and allografts using diffuse correlation spectroscopy. These findings represent an important step towards the assessment of graft blood flow to aid tissue engineering approaches developed to improve allograft healing and integration.

## Materials and Methods

### Ethics Statement

The study was conducted according to a protocol approved by the University Committee on Animal Resource (UCAR) at the University of Rochester (Permit number: UCAR-2010-056).

### Murine Segmental Femoral Graft Model

To assess the *in vivo* autograft and allograft healing processes, a previously established murine segmental femoral graft model was utilized in this study [4, 13, 14]. The graft transplantation procedure is summarized as follows: first, an osteotomy was performed to remove a 5 mm mid-diaphyseal segment of femur from the left hindlimb of a 6–8 week old female C57BL/6 mouse. Subsequently, a bone graft (either allograft or autograft) was transplanted into the bone defect and stabilized with the host bone using a 22-gauge intramedullary pin. For the autograft group, the femoral segment was removed and immediately transplanted back into the osteotomy. For the allograft group, the graft was prepared in advance by decellularizing the bone segment from a genetically different mouse strain (BALB/c) in a method described elsewhere [14]. Initially, 10 mice were prepared for each group. However, 3 mice in the autograft group did not survive the graft transplantation procedure due to the adverse effects associated with ketamine, anesthetic for the surgery. For post-surgical analgesia, 0.05 mg/kg buprenorphine was administered intraperitoneally on a daily basis for 3 days. Pain was monitored daily for 7 days. In cases where symptoms of pain persisted beyond 3 days post-surgery, additional buprenorphine was administered as needed until the symptoms subsided.

### Diffuse Correlation Spectroscopy Instrument

**Fig 1:**
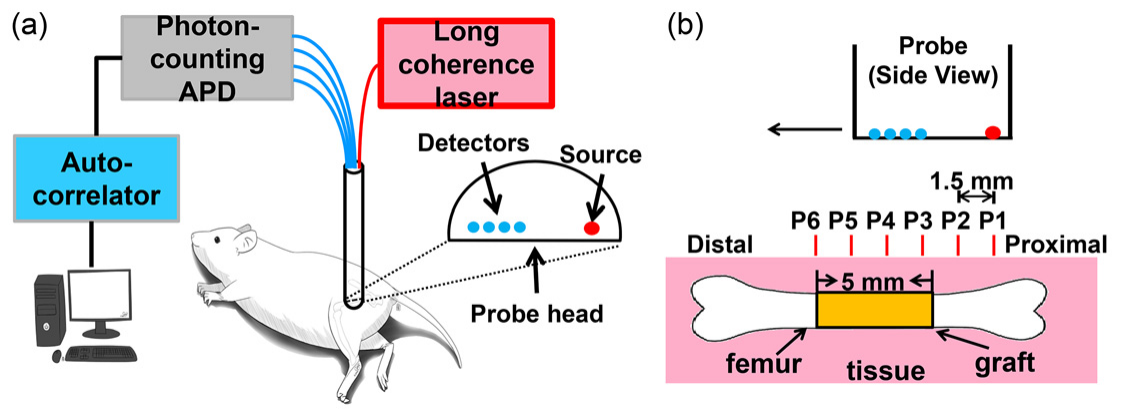
Instrument and probe placement. (a) Diffuse correlation spectroscopy system consists of a long coherence source, a contact probe, four photon-counting APDs (avalanche photodiodes) and an autocorrelator. (b) Measurement positions P1—P6 were chosen so that they were 1.5 mm apart along the femur starting from the proximal end. The source (red dot) and detectors (blue dots) in the probe were aligned along the femur.

A DCS system consists of a light source, a contact probe, four photon-counting detectors and a correlator as shown in Fig 1A. The light source is a 785 nm continuous wave, long-coherence-length (> 5 m) diode laser (DL785-120-SO, Crystal Laser, Reno, NV). A custom-made probe (FiberOptic Systems, Inc., Simi Valley, CA) containing optical fibers is placed in contact with the tissue of interest. A multi-mode fiber delivers near-infrared light from the source to the tissue and four single-mode fibers collect photons that have propagated through the tissue at different source-detector separations. The source-detector separations (SDs) are 2.9 mm, 3.6 mm, 4.3 mm and 5.0 mm respectively. Signals from different source-detector separations are then detected by four avalanche photodiodes (APDs, SPCM-AQ4C, Excelitas Technologies, Waltham, MA) and relayed to a 4-channel correlator board (Correlator.com, Bridgewater, NJ), which outputs the intensity temporal autocorrelation function.

### In Vivo DCS Measurement Protocol

The longitudinal measurements were performed on 7 mice in the autograft group and 10 mice in the allograft group. Measurements were performed one day before the graft surgery (week 0) to obtain the baseline blood flow, and then on a weekly basis for 9 weeks following graft implantation. The measurement protocol described below was designed to resolve spatial variations in blood flow in the graft leg and to follow global blood flow changes that affect the contralateral leg. The mice were anesthetized with continuous flow of isoflurane and oxygen and placed on a warming pad to maintain the body temperature. Hindlimb fur was shaved with clippers and the mice were positioned in a right recumbent position as shown in Fig 1A. DCS measurements were performed at six different positions along the femur on the left leg over the graft site. The muscle layer above the femur was about 1 mm thick in this right recumbent position. The source-detector separations used for measurements were 2.9 mm, 3.6 mm, 4.3 mm and 5.0 mm. The mice were then repositioned for a single mid-diaphyseal measurement on the right leg. Six locations designated as P1 to P6 are shown in Fig 1B, with P1 at the proximal end of the femur and P6 at the distal end. P7 refers to the measurement made on the contralateral (right) leg, 6 mm from the proximal end (corresponding to the mirror-image location of P5). The first position (P1) was determined by finding the proximal end of the femur by gentle palpation to identify the location of the greater trochanter. The probe was held by a micromanipulator mounted on a translational stage; the micromanipulator enabled stable and controlled vertical movements and the translational stage enabled horizontal movements. The probe was aligned such that the source and detectors were aligned parallel to the length of the femur, then lowered to make a gentle contact with the tissue. After the measurement, the probe was raised and translated 1.5 mm distally for subsequent measurements. At each position, 30 intensity autocorrelation functions were collected with an integration time of 2 seconds. After the experiment, the mouse was kept warm until it recovered from the anesthesia.

### DCS Data Analysis

To extract the tissue blood flow index *BFI*, the Nelder-Mead simplex method was utilized to fit the experimentally obtained autocorrelation function to the solution of correlation diffusion equation in a semi-infinite homogeneous medium [24]. Note that Brownian motion model of the moving scatterers was assumed. After extracting *BFI* from each autocorrelation function, the average *BFI* of 30 measurements collected at the same position was used to represent the blood flow. Relative blood flow (*rBF*) was defined by normalizing *BFI* at different time points with baseline *BFI* as the following:

$$rBF\;\left(r,t_{i}\right)=\frac{BFI\left(r,t_{i}\right)}{BFI\left(r,t_{0}\right)}$$ (1)

where *BFI*(**r**,*t*_*i*_) is the measured blood flow index at position **r** at the *i*th week after graft surgery and *BFI*(**r**,*t*_*0*_) is the baseline blood flow index at position **r** measured before the graft surgery.

To account for potential global blood flow changes associated with surgery and healing processes, and to separate out the local graft-specific blood flow changes, *rBF* difference between the graft leg and the contralateral leg (Δ*rBF*) was calculated for each mouse.

$$\Delta rBF\left(t\right)=rBF_{{}^{graft}}\left(t\right)-rBF_{{}^{contralateral}}\left(t\right)$$ (2)

The assumption here is that the blood flow changes on the graft leg are a linear combination of localized changes by the graft and global changes.

### Statistical Analysis

Two sample student’s t-test was carried out to compare the *rBF* of the graft leg and the contralateral leg in the same group or the *rBF* and Δ*rBF* of the autograft group and the allograft group in the same week. A *p*-value of less than 0.05 was considered statistically significant.

## Results

### Longitudinal Femoral Mid-Diaphyseal Blood Flow at Different Source-Detector Separations

**Fig 2:**
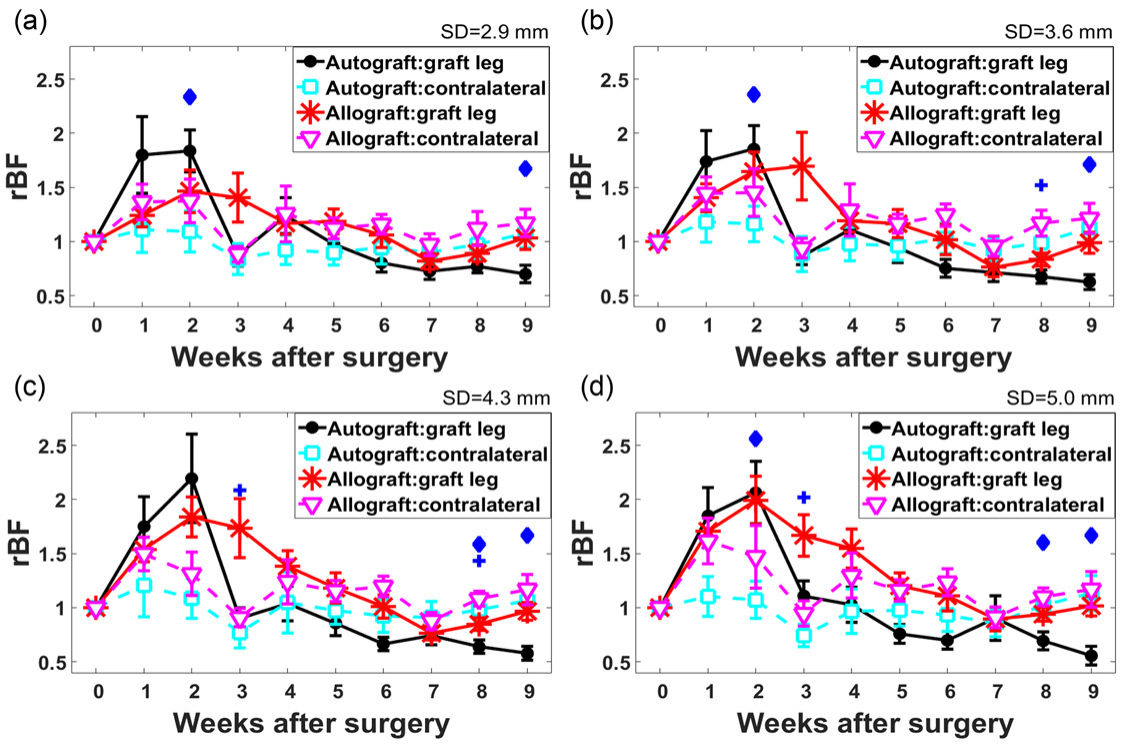
Longitudinal group-averaged relative blood flow (*rBF*)_changes at different source-detector separations (SDs). *rBF* changes during 9 weeks of measurement at P4 (graft leg) and P7 (contralateral control leg) for (a) SD = 2.9 mm, (b) 3.6 mm, (c) 4.3 mm and (d) 5.0 mm of autograft group (n = 7) and allograft group (n = 10). Each data point is the mean *rBF* of all the mice in the same graft group and the associated error bar is the standard error of the mean. The diamond sign denotes that *rBF* were significantly different between the graft leg and the contralateral control femurs in the autograft group at the corresponding week (*p* < 0.05). The plus sign denotes that *rBF* were significantly different between the grafted femur and the contralateral control femur in the allograft group at the corresponding week (*p* < 0.05).

Fig 2 shows the group-averaged longitudinal changes in relative blood flow (i.e., blood flow normalized to the pre-transplant time point) of the autograft group femurs (n = 7) and the allograft group femurs (n = 10) respectively. For the grafted femur, results are shown for position P4, which was located at the femoral mid-diaphysis. For the contralateral control femurs without any graft, data collected at position P7 are shown. Fig 2A–2D show data collected at SD of 2.9, 3.6, 4.3 and 5.0 mm respectively. The measurements at shorter source-detector separations are sensitive to superficial tissue, whereas the measurements at larger source-detector separations are sensitive to deeper tissue. Each data point represents the mean *rBF* of all mice in the corresponding group and associated standard error of the mean.

In the graft leg, mean *rBF* increased to more than 1.8-fold from the pre-transplant baseline during the first two weeks in the autograft group for all SDs. Subsequently, blood flow rapidly decreased to within 25% of baseline level (i.e., *rBF* = 1.25) at week 3. For the allograft group, mean *rBF* increased after the surgery and peaked at week 2 or 3. However, the maximum *rBF* was much greater at SD = 5.0 mm (maximum *rBF* = 1.99) compared to SD = 2.9 mm (maximum *rBF* = 1.46). Additionally, at SD = 5.0 mm, *rBF* did not decrease to within 25% of the baseline until week 5, while at SD = 2.9 mm *rBF* decreased at week 4.

In the contralateral control leg (no graft), mean *rBF* of the autograft group showed little variability (< 20%) throughout the 9 weeks of the study for all SDs. Interestingly, for the allograft group, the mean *rBF* of the contralateral control leg followed a similar trend to the grafted femur. For example, at SD = 5.0 mm a large increase in blood flow was observed: *rBF* = 1.61 and 1.47 at 1 week and 2 weeks after the surgery respectively. After week 2, the variation decreased (within 30%). A similar trend of increased blood flow in the contralateral control leg at other source detector separations was observed.

### Longitudinal Blood Flow Changes at Different Spatial Positions in Grafted Femurs

**Fig 3:**
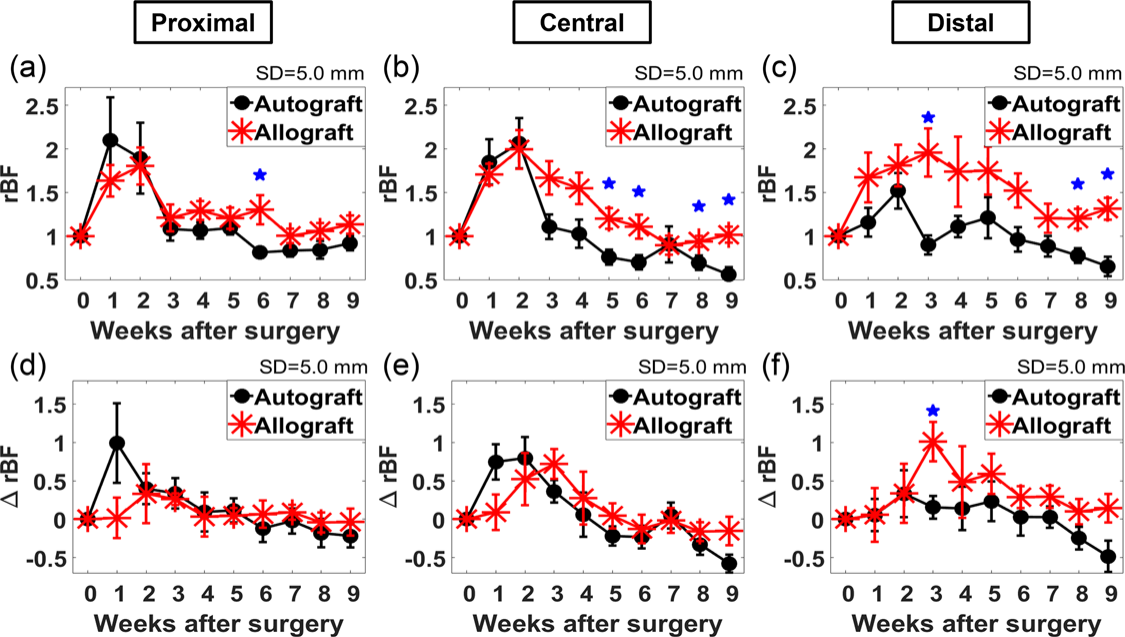
Longitudinal blood flow changes at proximal, central and distal part of the femur. Longitudinal relative blood flow (*rBF*) changes of the graft leg at (a) proximal (P2), (b) central (P4) and (c) distal (P6) locations are shown for the autograft (black circle) and allograft (red star) groups. Longitudinal changes in Δ*rBF* (graft-specific blood flow) at (d) proximal, (e) central and (f) distal locations are shown for the autograft (black circle) and allograft (red star) groups. Each data point is the group average and the associated error bar is the standard error of the mean. The pentagram denotes that the *rBF* or Δ*rBF* of two groups in the same week is significantly different (*p* < 0.05).

To compare the longitudinal blood flow changes at the bone graft and native bone segments, *rBF* changes at three different positions (P2, P4, P6) are shown in Fig 3A–3C. On the graft leg, position P2 represents the proximal part of the femur, position P4 represents the central part and position P6 represents the distal part (See illustration in Fig 1B). For clarity, only data at 5.0 mm SD are shown since they contain blood flow information from the greatest detection depth and capture the largest changes, as shown in Fig 2. In Fig 3A–3C, group-averaged *rBF* of the autograft group and allograft group are both shown. For the autograft group, the trend of *rBF* changes were similar at all three positions, i.e., elevated *rBF* were observed 1–2 weeks after graft transplantation and then *rBF* decreased to within 1.25-fold of the baseline at weeks3. The allograft group exhibited a spatially different pattern of *rBF* changes. Elevated *rBF* lasted for a shorter period in the proximal part compared to the distal part. The continuous time period during which *rBF* was 25% higher than baseline level was 2, 4, 6 weeks for proximal, central, and distal part respectively, which was 0, 2 and 4 weeks longer than that of the autograft group.

### Differences in Longitudinal Blood Flow Changes between Graft Femurs and Contralateral Control Femurs

In Fig 2, elevated *rBF* at weeks 1 and 2 were observed in both graft legs and contralateral control legs in the allograft group. To estimate graft-specific changes from overall changes including global changes, Δ*rBF*, the difference between *rBF* at different positions on the graft leg to *rBF* at the contralateral control femur (P7), were calculated and shown in Fig 3. Fig 3D–3F show Δ*rBF* at proximal (P2), central (P4), and distal (P6) locations along the femur respectively. Different patterns of longitudinal changes in Δ*rBF* were observed at different positions. At the proximal location, the difference in Δ*rBF* between the autograft group and allograft was small (< 25%) for all the time points except week 1. At the central location, for the autograft group, Δ*rBF* increased for the first two weeks and then started to decrease; for the allograft group, Δ*rBF* increased for the first three weeks and then started to decrease. The time for peak blood flow was delayed by one week in the allograft group compared to that in the autograft group. At the distal location, Δ*rBF* of the allograft group was significantly higher than that of the autograft group at week 3 (*p* < 0.05).

## Discussion

Most blood flow monitoring methodologies require animal sacrifice or provide a limited number of time points due to cost. Despite these limitations, results from these studies have informed our understanding of blood flow changes in fracture and interposition graft healing. The bone fracture healing process is similar to that of bone grafts [38], particularly for autografts since both the fracture model and autograft model involve autologous bone. Autologous bone healing can be divided into three distinct yet overlapping stages: (1) the early inflammatory stage; (2) the repair stage; and (3) the remodeling stage [38]. During the inflammatory stage (up to 7 days), a hematoma coagulates and infiltration of inflammatory cells and fibroblasts result in formation of granulation tissue, ingrowth of vascular tissue and migration of mesenchymal cells. The repair stage (1–3 weeks) involves new vasculature and bone callus formation at the fracture site, resulting in an increase in intravascular volume, vessel density, and bone callus volume at the graft site. In the remodeling stage, bone callus is resorbed and remodeled to restore the bone to its original shape, structure and mechanical strength, and the previously increased vascularity also regresses [38, 39]. In a rat fracture model using Laser Doppler flowmetry [22], blood flow was lower than the pre-fracture baseline immediately and one day after fracture, but returned to the pre-fracture baseline by day 3, and increased up to 40–80% greater than the baseline on day 7 or 14 depending on the spatial position. The lower blood flow in the early phase is expected due to disruption of bone vasculature from the injury. The subsequent increase of blood flow is probably due to inflammatory hyperemia. At 4 weeks post-fracture, blood flow returned to or slightly less than baseline level, which follows the expectation that the blood flow gradually returns to the pre-injury level during the remodeling stage [39]. Similarly Brueton *et al*. showed a longitudinal trend for blood flow with the peak occurring at day 14, whereas intravascular volume peaked at day 21 [40].

Studies have shown that autografts and allografts differ in temporal trends of vascular and bone callus volume. Autograft intravascular and bone callus volume peaks at 3 week post-implantation, whereas allograft vascular and bone callus volumes were still increasing 9 week post-implantation [13, 14]. Furthermore, autograft healing achieved pre-injury mechanical strength at 9 weeks whereas allografts exhibited significantly less strength than intact femurs after 16 weeks of healing [14]. These studies highlight the significant delay in healing of allografts in terms of intravascular volume and mechanical strength.

In our study, longitudinal blood flow changes during healing of autografts and allografts were quantified non-invasively with DCS using a murine segmental defect graft model. These measurements elucidated distinct differences in the spatiotemporal blood flow pattern in allografts compared to autografts in terms of (1) the relationship between the grafted and contralateral control femurs, (2) depth from the tissue surface, and (3) spatial locations along the femur.

Both grafts demonstrated a significant blood flow increase from the pre-transplant baseline at 1–2 weeks post-transplant, followed by a return to the baseline level. This transient increase in blood flow is likely due to several factors including inflammatory response to surgical insult, hyperemia, and subsequent angiogenesis required for tissue regeneration [38, 39], as described in the first paragraph of the discussion. In particular, autografts exhibited prolonged blood flow elevation for up to 2 weeks after surgery, similar to observations in a rat tibia fracture using laser Doppler flowmetry [22]. For the autograft group, blood flow of the contralateral femur did not change significantly from the baseline throughout 9 weeks. However, blood flow of the contralateral leg in the allograft group followed a similar trend of the femur with the graft, suggesting a more systemic inflammatory response to allograft placement.

Spatial differences in flow patterns between the autograft and allograft groups were also observed. Autografts yielded consistent post-transplant blood flow patterns both as a function of tissue depth and position along the graft (proximal to distal), while greater variation in blood flow patterns was observed in the allograft group as a function of depth and position. Results in Fig 3A–3C show a common pattern in blood flow changes at the proximal, central, and distal part of femur in autografts, similar to observations in fracture models in rats [22]. However, in the allograft group, the time for elevated *rBF* to return to baseline level was shortest for the proximal part and longest for distal part of the femur. In addition, proximal and central Δ*rBF* reached its peak earlier in the autograft group compared to the allograft group. Distal to the graft, the allograft group had a significantly increased Δ*rBF* compared to the autograft group at week 3, a trend that persisted for the duration of the experiment. Considering that autografts heal faster and more completely than allografts due to the presence of intact periosteum, delayed and prolonged elevation of Δ*rBF* may indicate delayed bone healing. Taken together, the presence of a global blood flow response and heterogeneous spatial and temporal blood flow patterns in the allograft group may be indicators of delayed healing. Further studies may be warranted to confirm these findings.

For the current study, there are three technical limitations that need to be considered in interpreting the results. First, DCS measurement provides bulk blood flow information including signals from both the bone and the surrounding soft tissues. The source-detector separation of 5 mm utilized in the current experiment is adequate to capture the optical signals from an organ located under 1 mm of overlying tissue. Due to the solid material aspect of bone tissue, the ratio of static and moving scatterers can be a concern. The significant amount of scattering from the static scatterers may affect the accuracy of the physical models used in diffuse correlation spectroscopy analysis that rely on randomization of the photon phases due to multiple scattering from moving scatterers [41]. However, since the photons travel through the surrounding soft tissues, which are well perfused, prior to reaching the bone and then they multiple scatter within the bone due to the high scattering nature of the bone [42], they are highly likely to contain a significant amount of signals from moving red blood cells (i.e., moving scatterers). Furthermore, since the bone is relatively well-vascularized [15], a considerable amount of this information comes from the bone itself. In fact, a previous study has shown the feasibility of using DCS to measure bone blood flow in human manubrium in a more extreme, larger geometry [43]. To distinguish the bone from the surrounding muscle, data analysis would require incorporation of a more sophisticated model to resolve flow as a function of depth. In the current analysis, solution to correlation diffusion equation for semi-infinite geometry was used. The recumbent position of the mice during the optical measurements flattened the tissue and aligned the femur in parallel to the ground, making the semi-infinite geometry assumption reasonable. Furthermore, the use of relative blood flow index, not the absolute blood flow index, minimized the inaccuracy caused by the simplified geometrical assumption (see below for more discussion). Note the use of relative blood flow is widely adopted in DCS brain applications [44]. Although not applied in this study, there are various advanced analysis methods to account for the tissue curvature and heterogeneity. These advanced methods feature a stand-alone or the combination of the following ideas: (1) use of measurements at multiple separations simultaneously to improve the stability and accuracy of DCS data fitting [45]; (2) use of analytical solution to layered model [46] or Monte Carlo simulation [47, 48] to account for the multiple-layer geometry; (3) use of large datasets for diffuse correlation tomography to reconstruct 3D distribution of blood flow [49, 50]. The second limitation is the difficulty in determining the precise graft position along the femur in the current experimental protocol. In the current DCS analysis, the center of the graft was assumed to be at the center of the femur. X-ray imaging of several mice (images not shown) revealed that the center of the graft could be shifted as much as 2 mm from the femur center. Similar temporal trends between P2 and P4 in Fig 3 may be due to this variability. To overcome these limitations, diffuse correlation tomography (DCT), which is an expansion of DCS to reconstruct the three dimensional blood flow, can be used to monitor the graft healing process. We have already demonstrated the feasibility of utilizing DCT for blood flow imaging of a murine hindlimb that has undergone allograft surgery and are in the process of further optimizing the technique [49]. The third limitation is the assumption that tissue optical properties, specifically absorption and scattering coefficients, are uniform in our region of interest when calculating blood flow index. The effect of errors in optical properties on blood flow index can be significant, with a reduced scattering coefficient being more influential towards blood flow index accuracy than the absorption coefficient [51]. While the quantification of absolute blood flow index can be significantly affected, we have demonstrated that relative blood flow normalized to the baseline largely mitigates these errors as long as the inherent tissue optical properties do not change significantly over time [49]. Assuming the graft is not grossly different from the original bone segment, the reduced scattering coefficient in this study can be assumed to be similar throughout the monitoring time period. However, the absorption coefficient may change significantly right after surgery. To estimate the degree of under/over-estimation of the absorption coefficient in the same experimental geometry and analysis scheme, numerical simulation data was generated using forward model in NIRFAST [52], on a mesh simulating a bone embedded in muscle, and fitted to an analytic solution to a homogeneous media at a source-detector separation of 5 mm. Allograft with no blood vessels would result in 10% underestimation of the absorption coefficient, which in turn results in 5% of blood flow index (*BFI*) error. This amount of error does not affect the overall trend observed in this study. In the future, diffuse optical spectroscopy/tomography measurements, a near infrared technique that can quantify tissue absorption and scattering, will be carried out along with DCS/DCT measurements to reduce the error as well as providing additional information on vascular volume and tissue oxygenation. Despite the current limitations, the difference in blood flow changes of the autografts and allografts were clearly observed in this study and we expect this difference will be more pronounced with future DCT measurements.

## Conclusion

In summary, longitudinal blood flow changes during autograft and allograft healing in a murine segmental defect model were monitored non-invasively with diffuse correlation spectroscopy. Our results show that blood flow within grafted femurs increased during the early weeks of healing and then started to decrease for both the autograft and allograft groups, similar to observations in a rat tibia fracture model using laser Doppler flowmetry [22]. The autograft group exhibited consistent patterns of spatial and temporal blood flow along the graft, with elevated blood flow only observed during the first 2 weeks before returning to the pre-operative baseline. For the allograft group, greater variations in patterns of blood flow were observed, as well as more prolonged periods of 2–4 weeks of elevated flow at the central and distal part of the femur. More specifically, Δ*rBF* in the autograft group, which is the graft specific blood flow change relative to the pre-transplant baseline, peaked 1 week earlier at the proximal and central part of the femur compared to the allograft group. However, at the distal end, the allograft group exhibited significantly higher Δ*rBF* than the autograft group (*p* < 0.05) at week 3. These differences in blood flow patterns may suggest delayed healing in allografts compared to autografts, as described elsewhere using post-mortem radiological and histological techniques [14].
